# The role of burden of disease assessment in tracking progress towards achieving WHO global air quality guidelines

**DOI:** 10.1007/s00038-020-01479-z

**Published:** 2020-10-15

**Authors:** Dimitris Evangelopoulos, Roman Perez-Velasco, Heather Walton, Sophie Gumy, Martin Williams, Frank J. Kelly, Nino Künzli

**Affiliations:** 1 Environmental Research Group, Imperial College, London, United Kingdom; 2 National Institute for Health Research Health Protection Unit: Environmental Exposures and Health, Imperial College, London, United Kingdom; 3 European Centre for Environment and Health, World Health Organization Regional Office for Europe, Bonn, Germany; 4 World Health Organization, Geneva, Switzerland; 5 Swiss Tropical and Public Health Institute (Swiss TPH), Basel, Switzerland; 6 University of Basel, Basel, Switzerland

**Keywords:** Air pollution, PM_2.5_, Burden of disease, Air quality guidelines

## Abstract

**Objectives:**

More than 90% of the global population live in areas exceeding the PM_2.5_ air quality guidelines (AQGs). We provide an overview of the ambient PM_2.5_-related burden of disease (BoD) studies along with scenario analysis in the framework of the WHO AQG update on the estimated reduction in the BoD if AQGs were achieved globally.

**Methods:**

We reviewed the literature for large-scale studies for the BoD attributed to ambient PM_2.5_. Moreover, we used the latest WHO statistics to calculate the BoD at current levels and the scenarios of aligning with interim targets and AQG levels.

**Results:**

The most recent BoD studies (2010 onwards) share a similar methodology, but there are differences in the input data which affect the estimates for attributable deaths (2.9–8.9 million deaths annually). Moreover, we found that if AQGs were achieved, the estimated BoD would be reduced by up to 50% in total deaths worldwide.

**Conclusions:**

Understanding the BoD across countries, especially in those that do not align with the AQGs, is essential in order to inform actions to reduce air pollution globally.

## Introduction

Air pollution is a major public health issue and a leading risk factor for mortality and morbidity worldwide (Cohen et al. 2017). It has been estimated to account for more than two-thirds of the environmental burden worldwide (Landrigan et al. 2018). More than 90% of the global population live in areas exceeding the World Health Organization global air quality guidelines (WHO AQGs) for particulate matter with diameter of less than 2.5 micrometres (PM_2.5_), i.e. 10 μg/m^3^ annual average (Shaddick et al. 2020). In 2004, WHO and the World Bank initiated the first comprehensive evaluation of ambient air pollution in the global burden of disease (GBD) study (Ezzati et al. 2004). Since then, many updates have been published, the most recent one by WHO reported that ambient and household air pollution contributed to 7 million deaths globally in 2016 (WHO 2018).

Over the last few years, numerous studies have combined evidence from exposure assessment and epidemiology in order to calculate the disease burden attributable to air pollution, using various methodologies. This has led to significant improvements in the burden of disease (BoD) methodology which have placed air pollution in the top tier of global risk factors in the public discussion. It has also driven important policy actions at country or global level with United Nations Sustainable Development Goals calling for substantial reductions in the ambient PM_2.5_-attributable deaths by 2030, an effort spearheaded by WHO (UN 2019). However, some have expressed concern that changes in estimates may come at the expense of an erosion of the public’s confidence in them (World Bank 2016). These discrepancies also have the potential of impacting public acceptance of interventions or hinder the identification of ‘true’ disease burden. Thus, it is important to regularly review data availability and methodologies and from an international or governmental body perspective, to balance the needs and assess what is best for policy purposes in terms of communication and capacity building support for resource-poor countries, especially in view of their growing interest on the subject matter.

The components of ambient air pollution that have been identified as major contributors to health deterioration (and quantified in BoD studies) are mainly PM_2.5_ and O_3_. These pollutants were identified as leading risk factors and the largest part of attributable mortality remains in low- and middle-income countries (LMICs) (GBD 2012, 2016, 2018). Other air pollutants, such as NO_2_, NO_x_, SO_2_, PM_10_ and CO, have also been associated with various adverse health effects. Only a few recent BoD studies have been conducted for some of these pollutants (Anenberg et al. 2018; Achakulwisut et al. 2019) since WHO published the context on using NO_2_ as a complementary pollutant for BoD (Héroux et al. 2015). However, while there are numerous studies that assess the BoD attributed to current levels of air pollution, very few report the potential reductions in the attributable mortality or gains in life expectancy if the 2005 WHO AQG levels or interim targets (ITs) were achieved globally (Apte et al. 2018; HEI 2019). The quantification of these reductions may support the ongoing update of the AQGs and assist authorities worldwide better understand the health benefits of reducing air pollution, including bridging the widening regional exposure inequality gap (Krzyzanowski and Cohen 2008). It might also inform policy makers and the public about the importance of lowering air pollution and the gain in public health that this reduction may cause.

This study was commissioned to provide WHO guideline development group (GDG) member information to be taken into account in the discussions of the new guidelines. The objectives of this paper are twofold. First, it provides the results of a WHO scenario analysis that estimates the extent to which BoD would be reduced if 2005 WHO AQGs and ITs were to be achieved in each WHO Member State, Region and globally. This simulation was conducted to support the guideline panel in their discussions about presenting ITs in the upcoming global AQGs. Second, it explains the differences in estimates from the ambient PM_2.5_-related BoD studies globally to provide context to the methods used in the scenario analysis and to improve general understanding of the various BoD estimates available.

## Methods

### History

The BoD associated with ambient particulate matter has been quantified using methods developed for an assessment requested by the Swiss Government (Künzli et al. 2000). These studies resulted in an adapted method applying PM_10_ for the first time in the WHO Comparative Risk Assessment/GBD Study (Ezzati et al. 2004; Cohen et al. 2005). Meanwhile, further developments by the Institute for Health Metrics and Evaluation (IHME) and GBD expert groups, using PM_2.5_ as the marker of pollution, became a default adopted by many local and regional studies. This approach estimates the proportional reduction in population disease or mortality that would occur if exposure to a risk factor was reduced to an alternative baseline level keeping other conditions unchanged. It combines information about the population exposure distribution, the exposure–response association and the best available morbidity and mortality data.

### Burden of disease overview

We searched the literature for large-scale studies for the BoD attributed to ambient PM_2.5_. The main aim of this overview was to summarize and compare the various inputs from these studies, including:

- Exposure assessment for ambient air pollution which combines (1) ground-level monitoring data, (2) estimates from remote sensing satellites and chemical transport models and (3) population, land use and topography information.
- The integrated exposure–response functions (IER) and more recent models to estimate the relative risk of cause-specific mortality over the global range of ambient annual mean PM_2.5_ concentrations.
- The counterfactual concentration for ambient air pollution assumed in their analysis and its distribution.
- The health outcomes that have been assumed to have an association with air pollution.

This overview was delivered to support the WHO GDG. Due to time constraints, we performed a selective review of the literature, focusing on studies that provide reference methods in the field from institutions, such as WHO and IHME. Some studies from independent researchers were also assessed, making sure that no great discrepancies in their inputs and outputs are observed.

### WHO scenario analysis

To explore the reductions in disease burden attributable to ambient PM_2.5_ globally, the current exposure levels were reduced to the current (2005) ITs, e.g. IT1 (35 μg/m^3^), IT2 (25 μg/m^3^) and IT3 (15 μg/m^3^) and AQG levels (10 μg/m^3^), using WHO data from 2016. The inputs and methods used for the burden estimates were those applied in the currently published WHO BoD estimates for 2016 (WHO 2018). In summary, exposure estimates were derived with the Data Integration Model for Air Quality (DIMAQ) model (Shaddick et al. 2018, available at https://www.who.int/airpollution/data/pm25_modelled_exposure_bycountry_2016_v0.xlsx?ua=1). Grid-level (11 km × 11 km) average exposure estimates were replaced, respectively, with ITs and guideline exposure level (GEL) when current levels were higher. Total number of deaths by country, sex and age group for ischaemic heart disease (IHD), cerebrovascular disease (stroke), chronic obstructive pulmonary disease (COPD), lung cancer and acute lower respiratory infection (LRI) were obtained from WHO Global Health Estimates 2018. Finally, the IERs used are those applied in GBD 2015 (available at https://cloud.ihme.washington.edu/index.php/s/puzbu28QteEHTmS). The calculations were based on a counterfactual ambient PM_2.5_ concentration between 2.4 and 5.9 μg/m^3^ (uniformly distributed), whereas the uncertainty in the attributable mortality estimates was calculated by matching random samples of each input variable. All calculations were done in *R*.

## Results

### Burden of disease overview

The reports by WHO and GBD are the most cited BoD projects (WHO BoD 2018; GBD 2018). However, other studies have been published reporting attributable deaths and disability-adjusted life-years (DALYs) (Burnett et al. 2018; Lelieveld et al. 2015; Silva et al. 2016). The basic principles of the methods used are mostly similar, but there are differences in the input values due to updated methodologies. For example, we report only the most recent BoD studies (from 2010 onwards) which assess PM_2.5_ exposures directly, while previous burden calculations converted PM_10_ estimates to PM_2.5_ using available information on geographic variation in the PM_2.5_/PM_10_ ratio (Cohen et al. 2005). Table 1 summarizes the key inputs and the findings of the most recent studies by WHO, the GBD study and approaches from independent researchers.

**Table 1:** Summary of inputs and outputs from various calculations of the global burden of disease from PM_2.5_, used as marker of ambient air pollution, ordered by publication date (reference column), for years 2010–2017 (this table is based on a selective review of the literature)

Year	References	Exposure assessment	Risk estimation	Counterfactual (μg/m^3^)	Cause of death	Deaths (95% UI) (millions)
2010	GBD (2012)	2005 data from ground-level monitors, remote sensing satellites and the CTM TM5 (0.1^o^ × 0.1^o^ resolution, Brauer et al. 2012)	IER findings from studies of air pollution, second-hand smoke and active smoking (8 studies, Burnett et al. 2014)	5.8–8.8 (uniform distribution)	LRI, lung cancer, COPD, stroke and IHD	3.2 (2.8–3.6)
2010	Lelieveld et al. (2015)	Global ECHAM5/MESSy atmospheric chemistry (EMAC)-general circulation model (1.1^o^ x 1.1^o^ resolution, Roeckner et al. 2006)	IER model (Burnett et al. 2014)	5.8–8.8 (uniform distribution)	LRI, lung cancer, COPD, stroke and IHD	3.2 (1.5–4.6)
2013	GBD (2015)	Same as GBD 2012 with 2011 data, an increased number of ground-level monitors and improved algorithms that incorporate uncertainty in the estimates (van Donkelaar et al. 2016)	Updated version of the IER model—(11 studies)	2.4–5.9 (uniform distribution)	LRI, lung cancer, COPD, stroke and IHD	2.9 (2.8–3.1)
2005	Silva et al. (2016)	Anthropogenic PM_2.5_ emissions from Mozart-4 (0.67^o^ x 0.5^o^ resolution, Emmons et al. 2010)	IER model (Burnett et al. 2014)	5.8–8.8 (uniform distribution)	Lung cancer, COPD, stroke and IHD	2.2 (1.0–3.3)
2012	WHO BoD (2016)	Hierarchical approach that combines data from ground-level monitors, satellites, CTM and other sources such as population, land use and topography—DIMAQ (Shaddick et al. 2018)	IER from GBD 2013 (updated version)	5.9–8.7 (uniform distribution)	LRI, lung cancer, COPD, stroke and IHD	3.0 (2.1–3.7)
2015	GBD (2016)	DIMAQ (as above)	New update of the IER model—(24 studies).	2.4–5.9 (uniform distribution)	LRI, lung cancer, COPD, stroke and IHD	4.2 (3.7–4.8)
2015	Burnett et al. (2018)	DIMAQ (as above)	GEMM NCD + LRI based on 41 cohorts (raw data from 15 cohorts) which models the shape of the CRF relaxing the assumptions of IER. Relies only on studies of outdoor PM_2.5_	2.4 (lowest observed concentration in any of the 41 cohorts)	Non-communicable diseases (NCDs) and lower respiratory infections (LRIs).	8.9 (7.5–10.3)
2016	WHO BoD (2018)	DIMAQ2 (updated to include within-country calibration variation)	IER from GBD 2015 (as above)	2.4–5.9 (uniform distribution)	LRI, lung cancer, COPD, stroke and IHD	4.2 (3.6–5.0)
2017	GBD (2018)	DIMAQ2	More recent update of the IER model	2.4–5.9 (uniform distribution)	LRI, lung cancer, COPD, stroke, IHD and diabetes	2.9 (2.5, 3.4)

*UI* uncertainty Interval, *CTM* chemical transport model, *DIMAQ* data integration model for air quality, *IER* integrated exposure–response functions, *GBD* global burden of disease, *LRI*: lower respiratory infection, *COPD*: chronic obstructive pulmonary disease, *IHD* ischaemic heart disease, *GEMM* Global Exposure Mortality Model, *NCD*: non-communicable diseases, *CRF* concentration–response function

Some of the key aspects regarding the similarities and the differences in the methodologies are discussed below:

- *Exposure assessment* There is an apparent agreement between recent studies regarding the choice of model used for deriving PM_2.5_ population-weighted annual average concentrations (GBD 2016; Burnett et al. 2018; WHO BoD 2018). DIMAQ provides estimates at spatial scales relevant to human exposure, using a hierarchical regression model under a Bayesian framework (Shaddick et al. 2018). It combines ground measurements from the WHO ‘Air pollution in cities’ database from more than 9000 monitors in 4300 cities globally, satellite data and chemical transport model predictions at approximately 11 km × 11 km spatial resolution (van Donkelaar et al. 2016) and population data from the GPW4 database (Center for International Earth Science Information Network 2016). Compared to models previously used in GBD studies (Brauer et al. 2016), DIMAQ showed improvements in fit and predictive ability. More specifically, R^2^, root mean square error (RMSE) and population-weighted RMSE all improved significantly resulting in values of 0.91, 6.6 μg/m^3^ and 12.1 μg/m^3^, respectively, for 2014 concentrations (Shaddick et al. 2018). However, no global estimates for the sources of the pollutant are available. Attributable mortality from major PM_2.5_ sources for China and India has been estimated previously, and similar work at a global level is underway (HEI 2016, 2018).
- *Risk estimation*: Analogous to the exposure assessment, most BoD studies are using IERs for each cause of death (*Burnett* et al. 2014). IERs are mathematical forms of the relative risk functions for various outcomes, informed by epidemiological studies of ambient air pollution, second-hand tobacco smoke, household use of solid fuel for cooking and active smoking to infer the shape of the function over the full range of global concentrations, including higher concentrations where epidemiological data were not available. In the presence of new evidence, updated IER versions have been available, and in GBD 2016, findings from 24 studies were combined to derive IERs for burden calculations. In GBD 2018, newly published cohorts were added for the construction of the IERs and Type II diabetes was included in the analysis. However, as mentioned above, IER pools risk estimates from studies of both ambient and non-ambient PM_2.5_, assuming equal toxicity per unit dose. Moreover, due to a lack of knowledge about the exposure–response relationship at low pollution levels, IER assumes a uniform distribution for the counterfactual value (see below). Burnett et al. (2018) tried to relax these assumptions and, by using individual data from 15 cohorts and pooled data from an additional 26 cohorts, developed the Global Exposure Mortality Model (GEMM). GEMM estimates a common hazard ratio for non-communicable diseases (NCD) and lower respiratory infections (LRI) over the range of ambient PM_2.5_ exposures observed in the 41 cohorts included, denoted GEMM NCD + LRI. The evolution and state-of-the-art methods used in the literature are extensively discussed in Burnett and Cohen (2020).
- *Counterfactual or theoretical minimum risk exposure level (TMREL)*: Because of the uncertainty regarding the adverse effects of low-level air pollution, researchers are using uniform distributions for TMREL based on information for the minimum and the fifth percentile of exposure distributions from outdoor air pollution cohort studies. Recent epidemiological evidence has shown adverse effects even at low levels (Brauer et al. 2019); thus, the values of the parameters for the uniform distributions used are decreasing over the years—from fixed values of 7.5 μg/m^3^ (for PM_2.5_ and PM_10_, Künzli et al. 2000; Ezzati et al. 2004), to 5.8–8.8 μg/m^3^ in *GBD 2012* and *WHO BoD 2016* and to 2.4–5.9 μg/m^3^ recently. Burnett et al. (2018) used a counterfactual value of 2.4 μg/m^3^ for PM_2.5_, which was the lowest observed concentration in any of the 41 cohorts included in their analysis. As the adverse effects seem to exist even at these low levels, the most recent counterfactual values seem to be reasonable.
- *Causes of death*: Up until 2017, BoD studies included the five health outcomes mentioned above, i.e. IHD (> 25 years), stroke (> 25 years), COPD (> 25 years), lung cancer (> 25 years) and LRI (all ages). Diabetes was included recently in the GBD air pollution assessment, and it was reported that it accounted for 184,000 deaths and 10.5 million DALYs globally (GBD 2018). The effects of air pollution on asthma in children, birthweight, preterm birth and cognitive function have been examined which might be included in future BoD studies. In particular, global BoD estimates of asthma attributable to NO_2_ have already been published (Anenberg et al. 2018; Achakulwisut et al. 2019).

Results from the GBD study and the WHO findings from 2016 are very similar due to similarities in the methods, apart from an update in the IER function and TMREL. The total number of deaths attributable to ambient air pollution is almost identical (4.2 million deaths), while there are only small differences in the cause-specific calculations. The male/female death ratio was higher in GBD 2016 compared to WHO BoD 2016, i.e. 1.38 vs 1.21, respectively (results not shown). Small differences were also observed in the estimated deaths by LRI and COPD (more LRI and less COPD deaths were estimated in WHO BoD 2018 compared to GBD 2016). On the other hand, in GBD 2018(where the exposure assessment changed as well) the total attributable mortality to ambient PM_2.5_ was significantly lower than in the two previous studies largely due to addressing previous double-counting of deaths attributable to ambient PM_2.5_ and PM_2.5_ from household use of solid fuel for cooking (2.9 million deaths). While not yet incorporated in the WHO or GBD reports, the estimated numbers substantially increased again in Burnett et al. (2018) due to the causes of death assumed (NCD + LRI) and the shape of the exposure–response association which deviates from the former defaults as it is steeper especially at the lower concentrations. They estimated that globally 8.9 million deaths were attributed to outdoor fine particulate matter. This number is 112% and 207% larger than GBD 2016, 2018, respectively.

### WHO scenario analysis

**Fig. 1:**
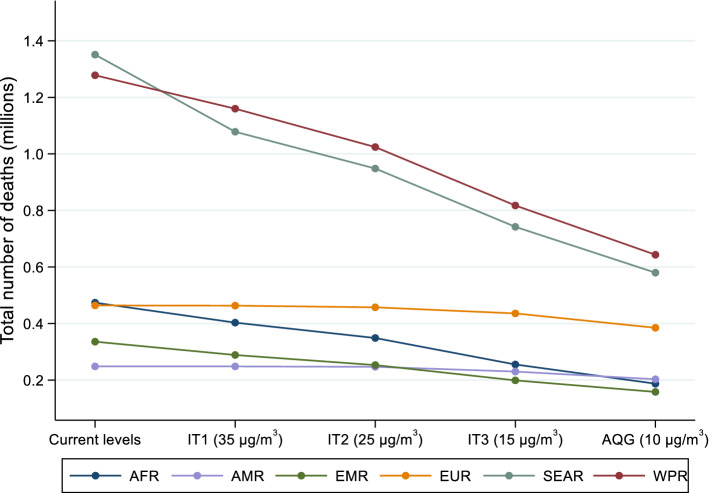
Reductions in the ambient PM_2.5_-attributable mortality by World Health Organization Region if the interim targets and guideline exposure values were achieved in 2016. *IT* interim targets, *AQG* air quality guideline, *AFR* African Region, *AMR* Region of the Americas, *EMR* Eastern Mediterranean Region, *EUR* European Region, *SEAR* South-East Asia Region, *WPR* Western Pacific Region

Table 2 and Fig. 1 illustrate the total number of deaths attributable to ambient PM_2.5_ by WHO region and worldwide. In all those scenarios, the indicated levels are assumed to reflect the population-weighted mean exposure. These attributable numbers were estimated using the current levels of exposure or the ITs and AQG levels. Even though the total number of attributable deaths for current levels of air pollution are much higher than those from GBD 2018 using 2016 data (2.8 million, HEI 2019), the distribution of deaths by WHO Region is quite comparable. Small differences are observed for the African and European Regions (10% and 12% of total deaths, respectively, compared to 6% and 16% in HEI 2019).

**Table 2:** Region-specific and global deaths attributable to ambient PM_2.5_ under the current (2016) levels of air pollution (with 95% uncertainty intervals (UI)) or if the interim targets and air quality guideline levels were achieved worldwide, assuming all other health relevant factors remained unchanged in 2016

Region			Global deaths and % reduction through achievement of IT or AQG level (95% UI)							
	Current (2016) Levels		IT1 (35 μg/m^3^)		IT2 (25 μg/m^3^)		IT3 (15 μg/m^3^)		AQG (10 μg/m^3^)	
	GBD estimates	WHO estimates	Deaths	% reduction	Deaths	% reduction	Deaths	% reduction	Deaths	% reduction
AFR	383,391 (329,623–439,917)	473,861 (410,387–546505)	403,371 (328,251–480,618)	14.5% (9.5%, 21.9%)	349,029 (269,680–429,497)	26.2% (17.4%, 37.0%)	255,339 (181,752–350,999)	45.9% (32.0%, 59.1%)	187,538 (126,465–284,393)	60.4% (44.0%, 72.0%)
AMR	256,170 (212,829–306,367)	248,530 (203,910–305,515)	248,530 (203,910–305,515)	0.0% (0.0%, 0.0%)	247,032 (201,932–304,015)	0.6% (0.4%, 0.9%)	230,302 (184,947–285,897)	7.4% (5.6%, 9.5%)	203,158 (158,744–257,521)	18.2% (14.4%, 22.5%)
EMR	350,281 (316,310–386,132)	335,958 (300,932–369,157)	288,838 (254,767–322,238)	13.8% (11.5%, 16.9%)	253,281 (220,088–287,453)	24.3% (20.4%, 28.9%)	199,164 (169,047–235,769)	40.4% (34.4%, 46.4%)	158,312 (129,824–193,704)	52.6% (45.7%, 58.9%)
EUR	513,890 (430,207–604,230)	464,172 (382,927–552,178)	463,395 (382,257–551,398)	0.2% (0.1%, 0.2%)	457,437 (376,375–545,234)	1.5% (1.2%, 1.9%)	435,954 (355,663–522,948)	6.2% (5.1%, 7.7%)	385,081 (307,856–471,386)	17.1% (14.2%, 20.4%)
SEAR	1,325,402 (1,154,834–1,501,356)	1,351,069 (1,193,394–1,515,015)	1,078,293 (939,631–1,244,190)	19.7% (16.3%, 25.1%)	948,327 (803,574–1,110,338)	29.5% (24.7%, 36.5%)	742,138 (609,868–906,467)	44.6% (38.0%, 52.8%)	579,943 (459,603–732,234)	56.8% (49.3%, 64.5%)
WPR	1,252,177 (1,099,708–1,416,942)	1,278,107 (1,118,969–1,449,439)	1,159,897 (1,009,363–1,324,466)	9.2% (7.9%, 11.2%)	1,024,245 (875,728–1,191,002)	19.8% (17.2%, 23.9%)	817,669 (673,349–977,601)	36.1% (31.7%, 42.5%)	643,432 (511,849–795,631)	49.7% (44.2%, 56.5%)
World	4,081,311 (3,543,511–4,654,944)^a^	4,154,936 (3,685,281–4,662,105)^a^	3,645,887 (3,179,495–4,188,266)	12.0% (9.7%, 15.5%)	3,271,578 (2,817,885–3,840,326)	20.8% (17.0%, 26.1%)	2,677,001 (2,237,067–3,221,859)	35.2% (29.4%, 42.3%)	2,155,152 (1,736,478–2,674,306)	47.8% (40.8%, 55.2%)

*IT* interim targets, *AQG* air quality guideline, *AFR* African Region, *AMR* Region of the Americas, *EMR* Eastern Mediterranean Region, *EUR* European Region, *SEAR* South-East Asia Region, *WPR* Western Pacific Region

^a^These values are slightly different than those presented in Table 1. This holds because the current levels differ slightly from the ones published earlier and reported in the 2016 WHO BoD and GBD 2016 due to rounding

Results from our scenario analysis show that if GEL had been achieved in 2016, the estimated BoD would have been reduced significantly, resulting in a 47.8% (uncertainty interval: 40.8–55.2%) decrease in total deaths compared with the current (2016) levels of exposure worldwide. The highest impact would be observed in the South-East Asian and African Regions (56.8% and 60.4% reduction, respectively). Meeting the ITs would also have a notable benefit on health, especially where exposures far exceed ITs. Even if IT1 was met, a 19.7% and 13.8% reduction in BoD attributable to ambient PM_2.5_ in the South-East Asian and Eastern Mediterranean Regions would have been observed.

**Fig. 2:**
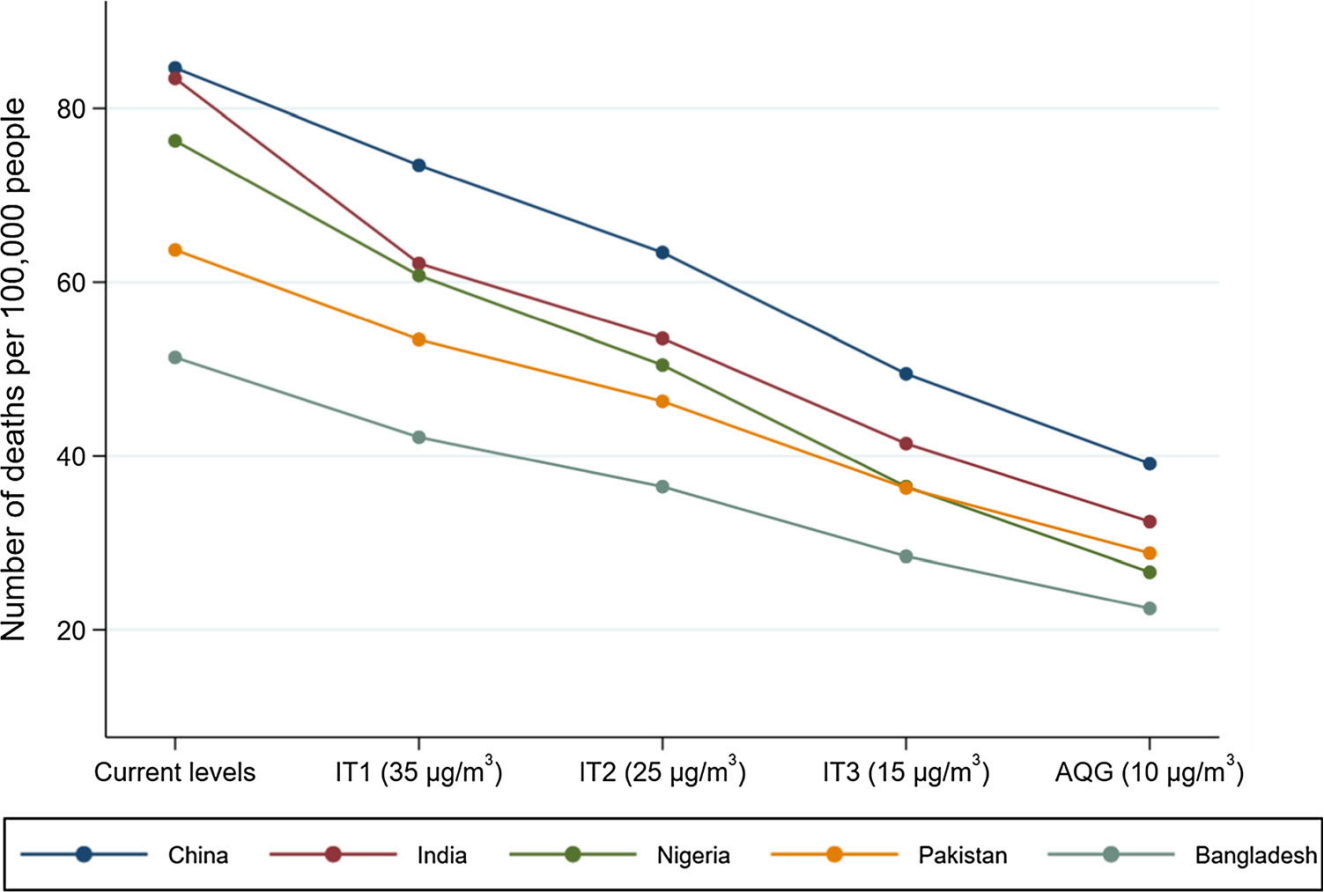
Reductions in the ambient PM_2.5_-attributable mortality if the interim targets and air quality guideline levels were achieved in five highly populated countries with high levels of air pollution, i.e. China, India, Nigeria, Pakistan and Bangladesh in 2016. *IT* interim targets, *AQG* air quality guidelines

The scenario analysis showed that if the ITs and GEL were achieved, the greatest benefit in BoD estimates would be observed in largely populated countries with high PM_2.5_ concentrations. Figure 2 shows the trend in the number of deaths per 100,000 people (for comparability) that would have been avoided for five countries with high levels of pollution (> 40 μg/m^3^) and the most estimated attributable deaths. India, Nigeria and China are the countries that would have experienced the greatest health benefit if they complied with the GEL, with a reduction of 51, 50 and 46 deaths per 100,000 people, respectively.

**Fig. 3:**
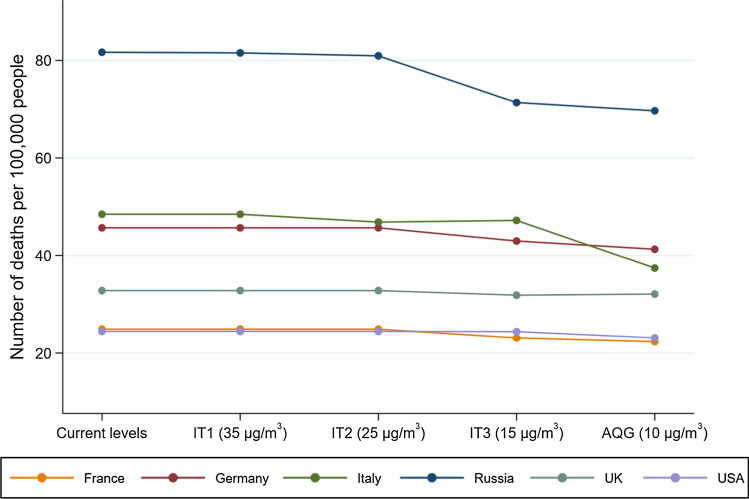
Reductions in the ambient PM_2.5_-attributable mortality if the interim targets and air quality guideline levels were achieved in the five highest populated European countries, i.e. France, Germany, Italy, Russian Federation, UK and the USA in 2016. *IT* interim targets, *AQG* air quality guidelines, *UK* United Kingdom, *USA* United States of America

On the other hand, results are different for higher-income countries, as in most cases PM_2.5_ in these areas is already below the ITs. Figure 3 illustrates the number of deaths that would have been avoided if the population-weighted average concentrations in the USA and the five most populous countries in Europe met the GEL (they already meet ITs). No difference is expected for the USA (modelled population-weighted mean exposure of 7 μg/m^3^) and only small changes in mortality for the United Kingdom, Germany and France. The alignment with the WHO GEL would have caused a higher drop in the deaths per 100,000 people in the Russian Federation (from 82 to 70) and Italy (from 48 to 37).

## Discussion

In this study, we provided an overview of the global burden of disease attributable to air pollution, i.e. the most important environmental risk factor worldwide (Landrigan et al. 2018), comparing the various methodologies and results available. Moreover, a working example was provided regarding WHO scenario analysis on the BoD reduction if 2005 WHO GEL and ITs had been achieved in 2016.

We summarized some recent studies that reported the estimated global BoD attributable to air pollution. Great discrepancies were found between them, i.e. from 2.2 to 8.9 million deaths attributable to ambient PM_2.5_ annually, because of the varying inputs and methods used. This variability is mainly due to different data and methods to estimate exposures, characterize exposure–response associations and quantify baseline rates of mortality in populations. Thus, the varying estimates regarding the number of people affected by air pollution are compared with reference to these different inputs.

Discrepancy in the estimates is a result of the combination of new data as well as updated methodologies, and it may sometimes cause loss of credibility and confusion among policy makers, governments and the public. Evolving estimates and methods, and choices thereof by institutions such as WHO need to be transparently communicated, at a level of technical detail suitable to the intended audience. These discrepancies are due to changes in population and demographic characteristics, true exposures and exposure assessment methods used and epidemiological evidence (Ostro et al. 2018; HEI 2019). Recent studies discuss the status of the current risk functions, identify the key uncertainties in the current GBD estimates and recommend solutions (Pope et al. 2018; Burnett and Cohen 2020; Shaffer et al. 2019). IHME and WHO provide a unified approach to exposure assessment for BoD estimation and have agreed to produce a single GBD Study with the aim of fully unifying methods in the spirit of the WHO/IHME collaboration *(*Tichenor and Sridhar 2019*)*. Yet, the critical assessment of the BoD methodology remains important for the development of strategies to reduce the impact of air pollution.

Despite the fact that air pollution has decreased in places that have implemented aggressive pollution controls (e.g. China), ambient PM_2.5_ continues to exceed the GEL and ITs (Shaddick et al. 2018). In GBD 2016 and WHO BoD 2018, it was estimated that 4.2 million deaths and more than 100 million DALYs were caused by ambient PM_2.5_. Taking into account the corresponding numbers for household air pollution and ambient ozone, but also other pollutants, such as NO_2_ (recently included in some BoD calculations despite issues related to the independence of effects (Héroux et al. 2015), one can conclude that air pollution is a leading mortality risk factor. Moreover, absolute numbers of attributable mortality have increased from 1990 to 2015 (even though mortality rates have declined), because of population growth, ageing, urbanization and increased pollution in some regions (Cohen et al. 2017; Ostro et al. 2018).

The scenario analysis showed that almost half of total deaths attributable to PM_2.5_ worldwide can be avoided if all countries comply with the GEL of 10 μg/m^3^. Regions with higher pollution levels, i.e. WHO South-East Asian, African and Eastern Mediterranean Regions, can benefit even more. Estimates showed that China, India, Nigeria, Pakistan and Bangladesh are the countries that would benefit most if they reach the ITs and GEL. The scenarios assumed an instantaneous exposure drop, whereas in normal conditions, reductions will occur over time. Full life-table analysis taking into account both partial reductions over time on the way to meeting the ITs and shifts in the size and age structure of the population as a result of the reductions would be more appropriate. However, this would be more time-consuming, and probably the comparisons across countries would be similar.

As mentioned, all our counterfactual scenario values are assumed to reflect the population-weighted mean exposure. However, the WHO AQGs call for compliance with the GELs at all locations where people live or work. A recent Swiss assessment included scenarios assuming compliance with GELs at all locations, including hot spots such as street canyons (Castro et al. 2020). Once this has been achieved, the population-weighted mean exposure is substantially lower than the GEL, i.e. average PM_2.5_ concentrations are expected to be 17% below the GEL once concentrations comply with the GEL at 99% of all locations. In the absence of estimates about the global population-weighted PM_2.5_ concentrations under full compliance with GELs, we have not included this approach. Thus, our results are probably a conservative estimate of the potential benefits of reaching GELs, even though GBD estimates are currently at sub-national level (11 km × 11 km resolution) with further estimates added in each update. This provides important future opportunities for analysis at finer scales, with recent studies showing great within-country variation for BoD estimates (Balakrishnan et al. 2019).

BoD calculations must be interpreted carefully because their methodology can be subject to limitations (Cohen et al. 2017; Shaffer et al. 2019). Recent evidence has shown that apart from cardiovascular and lung diseases, metabolic and other diseases are also associated with air pollution (Thurston et al. 2017). For such outcomes, only diabetes has been included in GBD 2018, so possibly we are underestimating the complete BoD attributable to air pollution. Moreover, the lack of exposure and health data and the absence of air pollution effect studies in most LMICs are another particular limitation. Given the different patterns of morbidities and risk factors prevailing in those countries, additional epidemiological research in those regions is warranted.

From a statistical perspective, BoD calculations use assumptions such as causality in the exposure–response association, the development of the IERs and the total adjustment for confounding. Causal inference in air pollution epidemiology (Nethery and Dominici 2019) and novel methods for the exposure–response models (Burnett and Cohen 2020) minimizing confounding bias can be used to better explain uncertainties in BoD methodologies.

Moreover, the exposures that are used for burden calculations are predictions from data integration models and can be subject to errors. While there is room for improvement especially in regions where monitoring is limited, e.g. WHO African and South-East Asian Regions, these models produce comprehensive sets of high-resolution exposure estimates (Shaddick et al. 2018). Such models can be used to identify areas with increased concentrations and greater uncertainty in the estimates, which can guide policy makers on future monitoring efforts.

Finally, our working example was on estimates for 2016, which are based on the most recent data available from WHO. The results might differ, although the magnitude is unlikely to change, if more recent inputs and methods were used. More specifically, the absolute number of deaths might be lower than estimated, e.g. if recent corrections of double-counting of deaths attributable to ambient and household PM_2.5_ were applied (GBD 2018). This does not necessarily imply that the scenario analysis percentage changes will differ as well. In addition, the scenarios tested in this analysis are based on ‘ideal’ situations in which PM_2.5_ levels drop to specific concentrations worldwide, while all the other factors (environmental or individual) that can act as confounders or effect modifiers in the PM_2.5_-mortality association remained unchanged. In normal conditions, this might not hold, and the exposure–response associations might be quite different from the current ones. However, results from studies at low levels of air pollution indicate that there is still an effect on health even at these levels (Héroux et al. 2015).

### Conclusions

It is imperative that work should be done in order to reduce air pollution globally. Towards this direction, WHO is updating AQG levels and interim targets. ITs have been defined as ‘incremental steps in progressive reduction of air pollution […] intended for use in areas where pollution is high’ (WHO Regional Office for Europe 2006). ITs are concentrations linked with health risk reductions, but using the relative risks of the exposure–response associations to communicate the health benefits can often be problematic. However, adding information on BoD results and transforming relative risks into number of deaths that could have been prevented can help guideline users to better understand the implications of reducing air pollution. Also, the investigation of various scenarios comparing BoD results using current levels of air pollution and target levels can increase awareness and inform governments to make decisions on more drastic measures. We plan to update our analysis when the new AQGs will become available using the most recent data and methods.

The BoD methodologies were originally developed to aid the prioritization of interventions in countries. In the context of AQGs, they can be used to indicate the number of deaths related to a certain risk reduction and assist users in designing air quality standards and policies, along with other considerations, such as the balance of the benefits and harms, resource implications, feasibility, equity and acceptability. However, understanding and reproducing the BoD across countries, especially in those that do not currently meet ITs and GEL, are essential in order to inform actions to reduce air pollution.
